# Triglyceride High-Density Lipoprotein Ratios Predict Glycemia-Lowering in Response to Insulin Sensitizing Drugs in Type 2 Diabetes: A Post Hoc Analysis of the BARI 2D

**DOI:** 10.1155/2015/129891

**Published:** 2015-05-27

**Authors:** Joel Zonszein, Manuel Lombardero, Faramarz Ismail-Beigi, Pasquale Palumbo, Suzy Foucher, Yolanda Groenewoud, Gary Cushing, Bernardo Wajchenberg, Saul Genuth, BARI 2D Study Group

**Affiliations:** ^1^Albert Einstein College of Medicine, Montefiore Clinical Diabetes Center, Bronx, NY 10461, USA; ^2^Department of Epidemiology, Graduate School of Public Health, University of Pittsburgh, Pittsburgh, PA 15260, USA; ^3^Case Western Reserve University and Cleveland VA Medical Center, Cleveland, OH 44106, USA; ^4^Mayo Clinic, Scottsdale, AZ 85259, USA; ^5^Montreal Heart Institute, Hotel-Dieu CHUM (Centre Hospitalier de l'Université de Montréal), Montreal, QC, Canada H2W 1T8; ^6^Toronto General Hospital, Toronto, ON, Canada M5G 2C4; ^7^Lahey Clinic Medical Center, Burlington, MA 01805, USA; ^8^University of Sao Paulo Heart Institute, 01246903 São Paulo, SP, Brazil; ^9^Case Western Reserve University, Cleveland, OH 44106, USA; ^10^University of Pittsburgh, Pittsburgh, PA 15260, USA

## Abstract

Glycemic management is central in prevention of small vessel and cardiovascular complications in type 2 diabetes. With the plethora of newer medications and recommendations for a patient centered approach, more information is necessary to match the proper drug to each patient. We showed that BARI 2D, a five-year trial designed to compare two different glycemic treatment strategies, was suitable for assessing different responses according to different phenotypic characteristics. Treatment with insulin sensitizing medications such as thiazolidinediones and metformin was more effective in improving glycemic control, particularly in the more insulin resistant patient, when compared to the insulin provision strategy using insulin and or sulfonylureas. Triglyceride and high density lipoprotein ratio (TG/HDL-cholesterol ratio) was found to be a readily available and practical biomarker that helps to identify the insulin resistant patient. These results support the concept that not all medications for glycemic control work the same in all patients. Thus, tailored therapy can be done using phenotypic characteristics rather than a “one-size-fits-all approach.”

## 1. Introduction

Information of long term clinical trials with combination glycemia-lowering therapy in type 2 diabetes mellitus (T2DM) is scarce. Recommendations for initial glycemic control consist of therapeutic lifestyle changes together with monotherapy, mainly metformin [1]. Personalization of medications should be done selecting the appropriate drug or drugs, ensuring patient decisions, quality of life, and balancing benefits of glycemic control with potential harm. Long term effectiveness data of monotherapy is limited to one clinical trial (ADOPT) comparing efficacy and side effects of three medications: metformin, glyburide, and rosiglitazone [2]. This study revealed differences among them and failure to normalize glycemia as long-term monotherapy, suggesting that combination therapy is necessary in the majority. With the plethora of newer medications available and recommendations for a patient centered approach, more information is necessary to match the proper drug to each patient [1]. The findings in this study support the concept that tailored therapy can be done according to the degree of insulin resistance, one aspect of the phenotype, rather than using the “one size fits all approach.”

The Bypass Angioplasty Revascularization Investigation 2 Diabetes (BARI 2D) clinical trial compared cardiovascular and diabetes treatment strategies with respect to mortality and cardiovascular events in patients with T2DM and stable CAD [3, 4]. It was a five-year trial designed to compare two different strategies, an insulin sensitization- (IS-) strategy primarily, where thiazolidinediones (TZDs) and/metformin were used, and an insulin provision- (IP-) strategy where insulin and/or a sulfonylurea (SUO) drugs were prescribed. We postulated that those patients with a greater degree of insulin resistance (IR) would respond better to an IS-strategy. Assuming this hypothesis was correct, tailoring therapy according to a patient's predominant pathogenetic phenotype would be more effective than using “a one drug fits all approach.” We reviewed patient characteristics and the impact on glycemic response to each of the two strategies, according to phenotype using dyslipidemia as a biomedical marker.

## 2. Methods

Detailed descriptions of BARI 2D have been published [3, 4]. With a 2 × 2 factorial design, the BARI 2D trial simultaneously assigned patients at random to one of two treatment strategies for glycemic control and to one of two revascularization strategies with all-cause mortality and cardiovascular events as outcomes. The diabetes component compared an IS-strategy that included TZDs and metformin versus an IP-strategy that included sulfonylureas (SUOs) and insulin. All patients were managed with aggressive medical therapy for hypertension, dyslipidemia, angina, and antiplatelet therapy. All blood tests were requested to be done in the fasting state; the glycemic and lipid values were affected by medical therapy. A HbA1c of <7.0% was the glycemic target, and patients were allowed to use medication from the opposite treatment arm if their HbA1c remained >8.0%. The second component of the 2 × 2 factorial design compared a strategy of prompt revascularization with aggressive medical treatment to a strategy of initial aggressive medical treatment alone with delayed revascularization if clinically required. Revascularization consisted of either percutaneous coronary intervention or coronary artery bypass graft (CABG) that was prespecified before randomization, with patients who had more severe CAD typically slated to undergo CABG.

In order to define higher degrees of IR we looked for practical clinical markers, a difficult task in an IR-population with type 2 diabetes that was already receiving intensive medical treatment. We determined the ability of the Adult Treatment Panel III (ATP III) criteria [5] where metabolic syndrome is present if three or more of the criteria are met. Since in the BARI 2D population all patients had diabetes and were receiving medical therapy, using this criterion was less than ideal. We therefore used triglycerides/high-density lipoprotein cholesterol (TG/HDL) ratios as a simple and practical surrogate marker for higher degrees of IR, a factor already validated in nondiabetic individuals [6]. A threshold of 3.75 in men and 3.00 in women was used to define IR.

A total of 2368 patients from 49 international sites were recruited. HbA1c was determined at baseline and at monthly and quarterly visits during the study follow-up. The duration of follow-up averaged 4.3 years. Of the 2368 patients, 2204 had baseline data to determine IR as defined above and also both a baseline HbA1c measurement and at least one follow-up value during or after the 6-month clinic visit. We identified a subset of 1308 patients whose baseline HbA1c was above the goal of 7.0%; they were designated as “likely candidates for glycemic improvement,” targeting this cohort for detailed analysis. Most participants were taking multiple prescription medications at study entry, with an average of 1.6 drugs for treatment of hyperglycemia, 2.2 drugs for angina, hypertension or heart failure, and 0.9 for lipids. Medications for glycemic control were extensively used with 54% taking metformin, 19% taking TZDs, 53% taking SUOs, and 28% taking insulin. Over 50% were treated with combination therapy.

We calculated the mean follow-up HbA1c for each patient as an “area-under-the-curve” (AUC) average, defined as the area under a line connecting the follow-up HbA1c values measured over time, divided by the length of time between the first HbA1c value starting at the 6-month visit and the last HbA1c value in the BARI 2D follow-up among 2204 patients who had HbA1c measurements available both at baseline and at 6 months or later. The multiple HbA1c values were summarized by calculating a single AUC mean per patient. For most patients, greater than >90%, each contributed somewhere between 5 and 25 HbA1c values to these calculations. The HbA1c values used in this analysis included those recorded in the central laboratory as well as those performed at the local sites. We use AUC mean rather than a conventional mean as the latter would be artificially high for patients who underwent repeated HbA1c tests during periods in which they were known or suspected to have poor glycemic control. Focusing on the 1308 patients who entered the study above goal (baseline HbA1c >7.0%), we used linear regression models to compare AUC average between patients randomized to an IP-strategy and patients randomized to an IS-strategy. The use of regression allowed to control for baseline HbA1c and also to do the comparison within groups defined by IR as defined above and also by other variables such as gender, age, and insulin use.

## 3. Results

**Figure 1:**
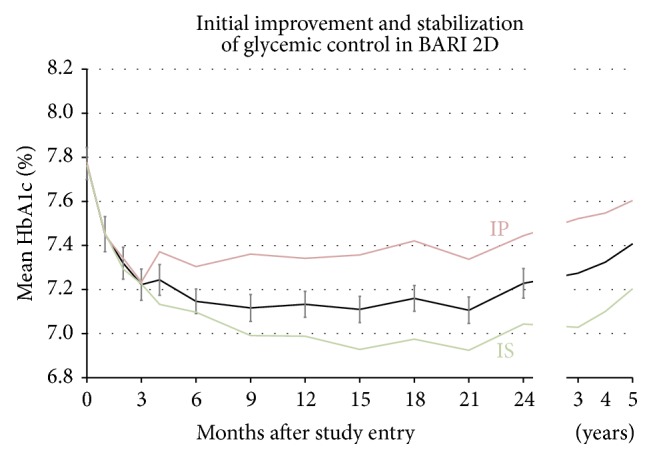
Estimates based on *n* = 2061 patients with HbA1c measurements at baseline, at 2 years, and at three or more follow-up visits in between. Shown are the mean response (black), IP-strategy (red), and IS-strategy (green).

**Figure 2:**
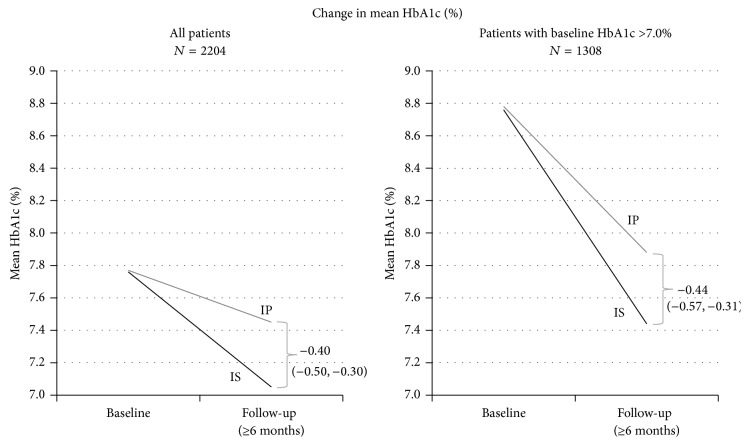
Improvement in glycemic control after randomization, overall and among the cohort of patients who entered the study with an HbA1c above goal (>7.0%).

Of the 2368 patients enrolled, 2061 had HbA1c measurements obtained at baseline, 2 years, and with three or more visits in between.  Figure 1 shows their mean HbA1c value, as measured by the participating clinical sites, starting at study entry and continuing during the protocol visits until the termination of the study. The patients were already under medical therapy, but after randomization and study entry, a rapid lowering of the mean HbA1c was observed between baseline (represented as month 0) and 3 months later. From a mean HbA1c of 7.8% at study entry, the individuals in the IS-strategy group experienced better glycemic control than those randomized to the IP-strategy, an unintended difference as the study tried to attenuate divergence between the two arms. The mean HbA1c remained fairly stable with some trend up at study end. After three months a significant separation between the IP-strategy and the IS-strategy can be noticed with improved glycemic control in the latter, a gap that remained throughout the duration of the study.  Figure 2 depicts the differences between IP and IS patients in glycemic control improvement following randomization. The mean follow-up HbA1c values are based on the individual (one per patient) AUC averages described earlier. The left panel illustrates changes of HbA1c in the entire population of 2204 patients and the right panel the changes among 1308 (59.3%) patients with a baseline HbA1c >7.0%. The higher baseline HbA1c resulted in a more dramatic improvement with IS patients with an overall reduction in mean HbA1c of 0.44% in excess of the reduction found in IP patients.

Baseline characteristics are summarized in Table 1. The analysis is largely focused on the cohort of patients with a baseline HbA1c of >7.0% (*n* = 1308). Using dyslipidemia measured as TG/HDL ratio as a surrogate for IR, the cohort was divided into 509 patients with a lower TG/HDL ratios (normal) and 799 patients with a higher TG/HDL ratios (elevated) and defined as having IR; 22 members of the latter group were treated with fibrates, niacin, or omega 3 polyunsaturates at baseline. The IR patients were more obese, younger, and with shorter duration of diabetes. There were no differences in gender, blood pressure, or blood pressure treatment at study entry. Differences were found among the ethnic/racial groups with a higher TG/HDL ratio more common in the non-Hispanic White and Hispanic populations, particularly those recruited in Mexico City. The non-Hispanic black population, on the other hand, had a lower TG/HDL ratio.

**Figure 3:**
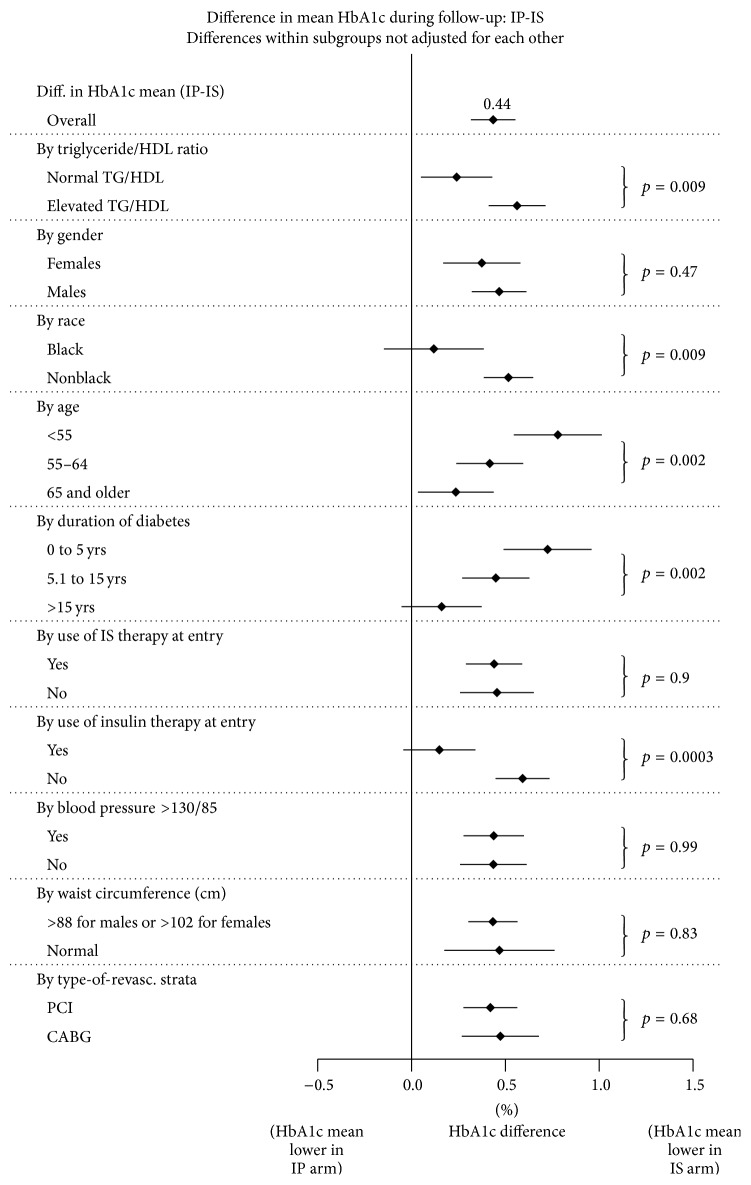
Overall and by-group differences in mean HbA1c between IP minus IS-strategies among patients with HbA1c >7.0 at baseline.

The degree of reduction in HbA1c in the IS versus the IP group was analyzed as a function of TG/HDL ratio is shown in Table 2. In the entire 2,204 patients analyzed, elevated TG/HDL ratio (≥3.75 in men, ≥3.00 in women) was associated with greater reduction of HbA1c (0.46% versus 0.29%; *p* = 0.05). In the 1308 patients who had HbA1c values of >7.0 at baseline, a greater reduction in HbA1c was observed in those with an elevated TG/HDL ratio (0.58% versus 0.24%; *p* = 0.009). The importance of the TG/HDL ratio as a marker for higher degrees of IR and increased responsiveness to IS medications is more evident in results shown in Figure 3. In the cohort of 1308 patients with HbA1c of >7.0% at baseline, increasing ratios in TG/HDL were associated with increasing decrements in HbA1c in the IS versus the IP group.

The TG/HDL ratio and other factors potentially associated with differences in the HbA1c levels between IP-strategies and IS-strategies during follow-up are also illustrated in Figure 3. In addition to the greater reduction in HbA1c in the IS group as function of TG/HDL ratio, better glycemic control was observed in younger patients and with shorter disease duration (*p* = 0.002). The improved response in the IS-strategy arm was more noticeable among non-Hispanic blacks (*p* = 0.008) and among patients who were not on insulin therapy at baseline (*p* = 0.0003). There was no difference by gender, blood pressure, waist circumference, and type of revascularization (*p* > 0.45 in all cases).

## 4. Discussion

Glucose control remains a major focus in the management of patients with diabetes, always in the context of a comprehensive cardiovascular risk factor reduction that includes smoking cessation, adoption of healthy lifestyle habits, blood pressure control, lipid management with priority to statin medications, and, when necessary, antiplatelet therapy. Recommendations for glycemic management are based on evidence-based clinical trial results performed in highly selected patient populations. In clinical practice these recommendations do not always address the range of choices available, nor do they address the vital question of which patient might respond better to which therapy and why, knowing patient-to-patient variability [7, 8].

We analyzed the impact of two different strategies targeting different pathogenetic factors with regard to glycemic management. The IP-strategy consisted in providing more insulin by increasing endogenous secretion through SU or exogenous insulin. The IS-strategy utilized metformin and a TZD aimed at reducing the IR that is commonly present in T2DM. The IS-strategy was superior in lowering HbA1c over the length of the trial, suggesting that IR, more than insulin deficiency, was perhaps the main driver of hyperglycemia. We then tested this hypothesis by using TG/HDL ratios as a biomarker of IR in a cohort of patients who were already under intensive medical management. More than half at study entry were treated with combination therapy, mainly metformin and SUOs and one-third were treated with insulin. From a mean HbA1c of 7.8%, the individuals treated according to the IS-strategy experienced better glycemic control, a difference that was unintended as the study attempted to attenuate this variance in order to compare these two arms on cardiovascular outcomes at similar HbA1c levels.

Preventing progression of ischemic heart disease was a major goal in BARI 2D and none of the two strategies had a definitive impact. Rosiglitazone was the most commonly used TZD drug, and despite findings suggesting that rosiglitazone increases the risk of cardiac ischemic events [9], this was not the case in the BARI 2D study, a high risk population with well-established CAD [3]. The two different strategies did not have an impact on the primary outcome of cardiovascular death or myocardial infarction. The divergence in glycemic response among the two strategies has clinical practice implications, suggesting that therapy can vary according to phenotypic characteristics enhancing a better response by choosing the right medication or combination of medications for each patient. This concept is particularly important nowadays with the widening array of pharmacotherapy to treat hyperglycemia [1].

Metformin remains the optimal drug of choice for monotherapy due to the low cost, proven safety record, weight neutrality, and possible benefits on cardiovascular outcomes. Little information is available regarding long term responses to the different medications and or identifying those who are better responders or no responders. Since monotherapy fails to achieve glycemic control in the majority, combination therapy appears to be necessary [2], but long term clinical trials outcomes of combination therapy are scarce. In addition to the BARI 2D, the only other randomized long term control study is the Rosiglitazone Evaluated for Cardiovascular Outcomes and Regulation of Glycaemia in Diabetes (RECORD) trial, designed to assess the effect of rosiglitazone on cardiovascular events. Rosiglitazone was used in combination with metformin, a SUO, or both, resulting in a lower mean HbA1c when compared to the control group at 5 years [10]. Treatment with rosiglitazone, however, increased heart failure risk and fractures mainly in women; the data while being inconclusive did not show increased cardiovascular morbidity or mortality risk when compared with standard glucose lowering drugs. In both RECORD and BARI 2D trials, sensitizers were more effective in reducing hyperglycemia.

Defining IR clinically remains a challenge, as insulin mediated glucose disposal is distributed continuously throughout the general population without absolute criteria with which to classify individuals as being IR. There is no optimal method to measure insulin resistance or insulin secretion in large clinical studies or in clinical practice. The convenient methods that have been proposed as suitable for large clinical studies have limitations, particularly when studying individuals with diabetes, with different etiologies of glucose dysregulation and or populations of diverse racial and ethnic backgrounds. Comparison of a patient's fasting values with the model's predictions allows a quantitative assessment of the contributions of insulin resistance and deficient *β*-cell function to the fasting hyperglycemia (homeostasis model assessment, HOMA); however, the low precision of the estimates from the model limits its use particularly in a diverse treated population with T2DM. Similarly, the oral minimal model remains cumbersome and will provide different values in different populations and be managed by different treatments.

Lipids and lipoprotein abnormalities are closely intertwined metabolically with insulin resistance and hyperglycemia. The combination of elevated TG, low HDL cholesterol, and relatively normal LDL cholesterol carried in small, dense, cholesterol-poor LDL particles has been known as diabetic dyslipidemia. People with obesity and insulin resistance have a characteristic dyslipidemia with an overproduction of very low density lipoproteins (VLDL) and hypertriglyceridemia [11–13]. The prevalence of obesity among diabetic patients and observations that plasma levels of free fatty acids are elevated suggests that free fatty acids are closely intertwined with glucose metabolism playing a role in insulin resistance and hyperglycemia [13]. Under normal physiological circumstances, fatty acids and hyperglycemia increase insulin secretion that offsets hyperglycemia by increasing muscle glucose uptake, inhibiting hepatic glucose output, and decreasing lipolysis. In IR individuals with T2DM the lack of compensatory insulin release causes a vicious cycle with worsening fatty acid-induced hyperglycemia [10–13]. Dyslipidemia, therefore, can be used as a biomarker of IR even in those individuals treated with “statins” (HMG-CoA reductase inhibitors), as they have an important reduction in LDL-cholesterol with a smaller impact in the TG/HDL ratio.

Having an easy-to-perform practical method of measuring insulin sensitivity can facilitate research, and ultimately one would hope it might help clinicians to better target personalized medicine for patients. Since the gold standard of IR are clamp studies which are not clinically practical, we used TG/HDL ratio as a readily available and useful biomarker of IR. A fixed threshold of TG/HDL ratio of 3.75 in men and 3.00 in women has been previously validated as a marker of IR in nondiabetic individuals [13], and the sensitivity and specificity are simpler and reasonably similar to the ATP III criteria used to define IR [14, 15]. The optimal TG/HDL ratio of 3.5 has a sensitivity and specificity that are comparable to the criteria proposed to diagnose the metabolic syndrome [6]. In BARI 2D, the majority of patients had dyslipidemia and the majority were already exposed to medications for glycemic and lipid management such as fibrates and or TZDs, agents that affect TG/HDL ratios but are not enough to completely reverse the dyslipidemia. Thus, the inclusion of individuals that were exposed to treatment most likely ameliorated the impact or severity of IR. Analysis of the degree of dyslipidemia using either quintiles or a fixed TG/HDL threshold correlated in a “dose related manner” with a better therapeutic response in the IS-strategy, making TG/HDL ratio a valuable and simple clinical tool. In summary, dyslipidemia, a component of IR, plays an important role in the underlying pathophysiology of hyperglycemia and can be used in the selection of mediations to treat hyperglycemia. Fixed TG/HDL ratio criteria vary among races with non-Hispanic black males having a lower TG and higher HDL concentrations than non-Hispanic white males [16], a disparity that is less pronounced among females [17]. Since non-Hispanic blacks have TG levels below the conventional threshold, the prevalence of IR using this parameter underrepresents this group [18, 19]. The non-Hispanic black population remains nonetheless IR and therefore TG/HDL ratio remains a useful indicator [18, 20]. Our study supports this discrepancy; nevertheless, glycemic control in the IS-strategy arm remained superior for the IR-non-Hispanic black population.

We found the IR-population in this study to be younger, with shorter disease duration and a higher HbA1c, as previously described [21]. These observations are relevant and consistent with the thought that T2DM has evolved into a newer and more aggressive disease affecting younger populations, with more central obesity, abnormal adipocytes, ectopic fat, and more dyslipidemia [22, 23]. The IR-population responded in a more robust manner to medications that target IR such as metformin and TZDs. As per protocol requirements, insulin was used in order to avoid HbA1c levels >7%. At the end of the study 78.5% of those randomized to the IP-strategy were on insulin, compared to 43.9% of those in the IS-strategy arm. The greater HbA1c response to IS, however, cannot be attributed to adding insulin, as it was used at twice the frequency in the IP-strategy. Insulin, necessary in the insulin deficient patient, is less effective in the more IR-population even when different regimens and higher doses are used. Due to the progressive *β*-cell dysfunction that characterizes T2DM, insulin may be necessary even when the individual appears to be IR [24]. The IP-strategy is more effective in the less IR-population with longer disease duration, suggesting that insulin deficiency is the major driver of hyperglycemia.

Preventing progression of ischemic heart disease was a major goal in BARI 2D and rosiglitazone was the most commonly used TZD drug in the study (donated by GlaxoSmithKline). Due to restrictions on rosiglitazone use by the Food and Drug Administration requiring submission of a Risk Evaluation and Mitigation Strategy (REMS), pioglitazone remains now the only TZD prescribed and found to be beneficial when added to existing treatment [25, 26]. While pioglitazone is an effective insulin sensitizer, its use needs to be carefully weighed against side effects that include risk of bone fractures, weight gain, edema, and increased incidence of heart failure, particularly when prescribed at high doses or when used in combination with SUOs or insulin [26–28].

Effective treatment of T2DM requires multiple drugs used in combination to correct the multiple pathophysiological defects, should be started early and based on pathogenic abnormalities and not simply on the reduction in HbA1c [29]. However, with a distinct paucity of long term comparative-effectiveness trials, recommendations on the best combination regimens remain elusive [30]. Data will be generated from a combination trial now in progress, a study comparing adding either sitagliptin, liraglutide, glimepiride, or insulin to metformin therapy [31]. In the meantime, findings from BARI 2D support the concept that individuals with IR better respond to an IS-regimen and demonstrate that tailored therapy for glycemic control is plausible. Since the BARI 2D trial, newer medications have been introduced and more long term data regarding the cardiovascular impact of glucose lowering therapies will become available. The future looks promising and management of diabetes during the past decade has already resulted in improved cardiovascular disease risk outcomes [32].

*Strengths and Limitations*. The major strength of this study is that it was randomized, prospective, and in a large population. It provided information of the phenotypic characteristics of a selective population with T2DM and stable CAD. It also offered information on the effect of glycemic control using two different strategies of combination therapy in a specific population. While we were successful in studying a diverse ethnic/racial populations, the non-Hispanic black population was singular in that the criteria used for IR based on dyslipidemia while being effective were less reliable. Using lipid parameters to predict IR in overweight individuals of other racial/ethnicities is a limitation in this post hoc analysis. Moreover, metabolic abnormalities may differ among racial groups. A further limitation is that the study was performed using “older” agents and many of the newer medications that have different glycemic impact were not studied.

## 5. Conclusions and Practice Changes

The results of our study confirm that BARI 2D was suitable for assessing different phenotypic characteristics and dissimilar outcomes according to different interventions. The use of TG/HDL-cholesterol ratios represents a simple clinical tool that may help identify the IR patient and could help tailor diabetes therapies. Treatment with IS-strategies in the IR-population appears to be more beneficial in reducing glycemia. It is imperative that properly planned randomized long term control studies using combination therapy are performed in order to establish best practices in treating the ever expanding IR-population with T2DM.

## What Is Already Known on This Topic?

 Coronary artery disease is common in type 2 diabetes population with the dyslipidemia of diabetes.
 Dyslipidemia of diabetes characterized by elevated triglycerides and low high-density lipoprotein-cholesterol is commonly associated with insulin resistance.
 Glycemic control is suboptimal in patients with type 2 diabetes, particularly those with insulin resistance.

## What This Study Adds

 It provides phenotypic characteristics of patients with type 2 diabetes and coronary artery disease and shows a high prevalence of the metabolic syndrome risk factors.
 Insulin sensitizing therapy is more effective than insulin providing therapy in achieving glycemic control in patients with type 2 diabetes and coronary artery disease.
 The use of triglycerides/high-density lipoprotein cholesterol ratio is a simple surrogate to help identify individuals that achieve better glycemic control with use of insulin sensitizers.
 Response to medications for glycemic control differs among patients and they can be chosen according to pathophysiology or phenotypic characteristics rather than using a “one size fits all approach.”

## Figures and Tables

**Table 1:** Baseline characteristics for the cohort with HbA1c >7.0%.

Baseline characteristics	All TG/HDL ratios (*N* = 1308)	Normal TG/HDL ratio^*∗*^ (*N* = 509)	Elevated TG/HDL ratio^*∗*^ (*N* = 799)	Normal versus elevated *p* value
Female	33.3	31.8	34.3	

Race/ethnicity, %				<0.0001
White non-Hispanic	61.2	51.1	67.6	
Black non-Hispanic	19.6	28.9	13.8	
Hispanic	13.7	13.0	14.1	
Asian non-Hispanic	5.0	6.7	4.0	
Other non-Hispanic	0.5	0.4	0.5	

Region of world, %				0.006
USA	60.2	62.9	58.4	
Canada	15.1	16.9	13.9	
Mexico	4.7	2.9	5.9	
Brazil	16.9	15.5	17.8	
Czech Republic/Austria	3.1	1.8	4.0	

Age at study entry, mean, SD	61.1, 8.8	62.3, 8.5	60.4, 8.9	0.0002

Duration of DM, mean, SD	11.7, 8.7	13.0, 9.5	10.9, 8.1	<0.0001

HbA1c %, mean, SD	8.8, 1.5	8.6, 1.4	8.9, 1.4	0.002

On insulin, %	35.9	40.3	33.2	0.009
On sulfonylurea, %	56.9	52.5	59.8	0.009
On metformin, %	56.8	53.4	59.0	0.047
On TZD, %	18.5	20.8	17.0	0.09
On either metformin or TZD, %	63.1	60.3	64.9	0.09

High BP (>130/85), %	54.9	55.7	54.3	
On ≥2 BP medications, %	76.8	75.9	77.3	
Uncontrolled BP on medications, %	43.0	44.2	42.2	

Waist circumference cm, mean, SD	107.3, 13.8	105.7, 14.4	108.4, 13.3	0.0006
BMI, mean, SD	31.7, 6.0	31.2, 6.5	32.0, 5.6	0.03

TG mg/dL, mean, SD	188.9, 143.5	97.7, 34.3	247.0, 155.8	<0.0001
HDL mg/dL, mean, SD	38.5, 10.3	44.7, 11.1	34.6, 7.5	<0.0001
TG/HDL, mean, SD	5.64, 6.29	2.28, 0.84	7.79, 7.25	

On statins, %	73.2	75.8	71.6	0.09
On fibrates, %	8.0	0.0	13.0	
On niacin, %	1.5	0.0	2.4	

CABG-STRATA, %	32.9	30.3	34.5	

^*∗*^Elevated triglyceride/high-density lipoprotein cholesterol (TG/HDL) ratio means having a value of triglyceride (mg/dL) divided by HDL-cholesterol (mg/dL) that is 3.75 or higher in men or 3.0 or higher in women. All others are considered “normal.” Note that only 777 patients had TG/HDL ratio at baseline that was actually elevated. An additional 22 patients are included in this group because they were treated for dyslipidemia (fibrate, niacin, or omega 3 polyunsaturates).

**Table 2:** Analysis of HbA1c and differences in the response to therapy evaluated on all patients, on patients above goal at baseline (HbA1c > 7.0%).

Categorizations	All			Above goal (baseline HbA1c > 7.0%)		
	*N*	HbA1c difference: IP minus IS Estimate (95% CI)	Homogen tests *p* values	*N*	HbA1c difference: IP minus IS Estimate (95% CI)	Homogen tests *p* values
Overall	2204	0.39 (0.31, 0.47)		1308	0.44 (0.32, 0.55)	

TG/HDL fixed ratio groups^*∗*^			0.05			0.009
Normal TG/HDL	879	0.29 (0.16, 0.42)		509	0.24 (0.05, 0.43)	
Elevated TG/HDL	1325	0.46 (0.35, 0.56)		799	0.56 (0.41, 0.71)	

TG/HDL ratio by categories^*∗∗*^			0.65 (trend: 0.12)			0.18 (trend: 0.01)
TG/HDL < 2	329	0.31 (0.10, 0.52)		188	0.23 (−0.08, 0.55)	
TG/HDL ≥ 2 and <3	354	0.30 (0.10, 0.51)		203	0.27 (−0.03, 0.57)	
TG/HDL ≥ 3 and <4	344	0.39 (0.18, 0.59)		211	0.35 (0.06, 0.64)	
TG/HDL ≥ 4 and <5	224	0.49 (0.23, 0.74)		134	0.50 (0.13, 0.87)	
TG/HDL ≥ 5	953	0.44 (0.32, 0.57)		572	0.60 (0.42, 0.78)	

^*∗*^Elevated triglyceride/high-density lipoprotein cholesterol (TG/HDL) ratio means having a value of triglyceride (mg/dL) to HDL-cholesterol (mg/dL) that is 3.75 or higher in men or 3.0 or higher in women. All others are considered “normal.”

^*∗∗*^Categories of triglyceride/high-density lipoprotein cholesterol (TG/HDL) ratio, divided arbitrarily into 5 groups, from <2 (least) to ≥5 (most) insulin resistance.

## Appendix

### The BARI 2D Study Group

University of Pittsburgh, Pittsburgh, PA (Coordinating Center): (Principal Investigator) Katherine M. Detre, MD, DrPH (deceased), Sheryl F. Kelsey, PhD (Co-Principal Investigator) Maria Mori Brooks, PhD (Coinvestigators) Trevor J. Orchard, MBBCh, MMedSci, Stephen B. Thomas, PhD, Kim Sutton Tyrrell, RN, DrPH (deceased), Jamal S. Rana, MD, PhD (Coordinator) Frani Averbach, MPH, RD (Administrative Coordinators) Joan M. MacGregor, MS, Scott M. O'Neal, MA, Kathleen Pitluga, BA, Veronica Sansing, PhD, Mary Tranchine, BS, Sharon W. Crow, MEd (Statisticians) Marianne (Marnie) Bertolet, PhD, Regina Hardison, MS, Kevin Kip, PhD, Manuel Lombardero, MS, Jiang Lu, MS (Data Managers) Sue Janiszewski, MSIS, Darina Protivnak, MSIS, Sarah Reiser, BS (System Programmers) Stephen Barton, ASB, Ping Guo, BS, Yulia Kushner, BS, BA (System Support) Jeffrey P. Martin, MBA, Christopher Kania, BS, Michael Kania, BS, Jeffrey O'Donnell, BS (Consultant) Rae Ann Maxwell, RPh, PhD, Mayo Clinic Foundation, Rochester, MN: (Study Chair) Robert L. Frye, MD National Heart, Lung, and Blood Institute, Bethesda, MD, National Institutes of Health, Bethesda, MD (Program Office): (Project Officer) Suzanne Goldberg, RN, MSN (Deputy Project Officer) Yves Rosenberg, MD, MPH (NHLBI Officers) Patrice Desvigne-Nickens, MD, Abby Ershow, ScD, David Gordon, MD, PhD, Dina Paltoo, PhD, MPH National Institute of Diabetes and Digestive and Kidney Diseases, National Institutes of Health, Bethesda, MD (Cofunding): (Program Director for Diabetes Complications) Teresa L. Z. Jones, MD University of São Paulo Heart Institute, São Paulo, Brazil (Clinical Site): (Principal Investigators) Cardiology: Whady Hueb, MD, José Ramires, MD, Neuza Lopes, MD; Diabetology: Bernardo Léo Wajchenberg, MD (Investigators) Eulogio E. Martinez, MD, Sergio A. Oliveira, MD, Expedito E. Ribeiro, MD, Marcos Perin, MD (Coordinator) Roberto Betti, MD Toronto General Hospital/University Health Network, Toronto, Canada (Clinical Site): (Principal Investigators) Cardiology: Leonard Schwartz, MD; Diabetology: George Steiner, MD (Investigators) Alan Barolet, MD, Yolanda Groenewoud, MD (Coordinators) Lisa Mighton, RN, CDE, Kathy Camelon, RD, CDE Texas Health Science at San Antonio/South Texas Veterans Health Care System, San Antonio, TX (Clinical Site): (Principal Investigators) Cardiology: Robert O'Rourke, MD (deceased); Diabetology: Janet Blodgett, MD (Investigators) Edward Sako, MD, PhD (Coordinators) Judith Nicastro, RN, Robin Prescott, MSN Mayo Clinic, Rochester, MN (Clinical Site, Vanguard Site): (Principal Investigators) Cardiology: Charanjit Rihal, MD; Diabetology: Frank Kennedy, MD (Investigators) Gregory Barsness, MD, Amanda Basu, MD, Alfredo Clavell, MD, Robert Frye, MD, David R. Holmes Jr., MD, Amir Lerman, MD, Charles Mullaney, MD, Guy Reeder, MD, Robert Rizza, MD, Hartzell Schaff, MD, Steven Smith, MD, Virend Somers, MD, Thoralf Sundt, MD, Henry Ting, MD, R Scott Wright, MD (Coordinators) Pam Helgemoe, RN, Diane Lesmeister, Deborah Rolbiecki, LPN Mexican Institute of the Social Security, Mexico City, DF, Mexico (Clinical Site): (Principal Investigators) Cardiology: Luis Lepe-Montoya, MD (deceased); Diabetology, Jorge Escobedo, MD, FACP (Investigators) Rafael Barraza, MD, Rubén Baleón, MD, Arturo Campos MD, Paula García, MD, Carlos Lezama, MD, Carlos Miramontes, MD, Salvador Ocampo, MD, Joaquín V. Peñafiel, MD, Aquiles Valdespino, MD, Raúl Verdín, MD, Héctor Albarrán, MD, Fernando Ayala, MD, Eduardo Chávez, MD, Héctor Murillo, MD (Coordinators) Luisa Virginia Buitrón, MD, Beatriz Rico-Verdin, MD, PhD, Fabiola Angulo, CCT University Hospitals of Cleveland/CASE Medical School Cleveland, OH (Clinical Site, Vanguard Site): (Principal Investigators) Cardiology: Dale Adler, MD, Austin Arthur Halle, MD; Diabetology: Faramarz Ismail-Beigi, MD, PhD (Investigators) Suvinay Paranjape, MD (Coordinators) Stacey Mazzurco, RN, Karen Ridley, RN, BSN Memphis VA Medical Center/University of Tennessee, Memphis, TN (Clinical Site): (Principal Investigators) Cardiology: Kodangudi Ramanathan, MD; Diabetology: Solomon Solomon, MD, Nephrology: Barry Wall, MD (Investigators) Darryl Weinman, MD (Coordinators) Tammy Touchstone, RN, BSN, Lillie Douglas, RN Montréal Heart Institute/Hôtel-Dieu-CHUM Montréal, Canada (Clinical Site, Vanguard Site): (Principal Investigators) Cardiology: Martial Bourassa, MD, Jean-Claude Tardif, MD; Diabetology: Jean-Louis Chiasson, MD, Marc Andre Lavoie, MD, Rémi Rabasa-Lhoret, MD, PhD (Coordinators) Hélène Langelier, BSC, RD, Suzy Foucher, RN, BA, Johanne Trudel, RN, BSc Albert Einstein College of Medicine/Montefiore, Bronx, NY (Clinical Site): (Principal Investigators) Cardiology: Scott Monrad, MD, Vankeepuram Srinivas, MD (deceased); Diabetology: Joel Zonszein, MD (Investigators) Jill Crandall, MD (Coordinators) Helena Duffy, ANP, CDE, Eugen Vartolomei, MD Fuqua Heart Center/Piedmont Hospital, Atlanta, GA (Clinical Site): (Principal Investigators) Cardiology: Spencer King III, MD, Carl Jacobs, MD; Diabetology: David Robertson, MD (Coordinators) Marty Porter, PhD, Melanie Eley, RN, CCRC, Emmalee Nichols, BS, CRC, Jennifer LaCorte, RN, BSN, CCRN, Melinda Mock, RN, BSN, MA University of Alabama at Birmingham, Birmingham, AL (Clinical Site, Vanguard Site): (Principal Investigators) Cardiology: William Rogers, MD; Diabetology: Fernando Ovalle, MD, David Bell, MBBCh (Investigators) Vijay K. Misra, MD (deceased), William B. Hillegass, MD, Raed Aqel, MD (Coordinators) Penny Pierce, RN, BSN, Melanie Smith, RN, BSN, Leah Saag, RN, Ashley Vaughn, RN, Dwight Smith, RN, Tiffany Grimes, RN, Susan Rolli, RN, Roberta Hill, RN, Beth Dean Barrett, RN, Clarinda Morehead, LPN, Ken Doss Northwestern University Medical School, Chicago, IL (Clinical Site): (Principal Investigators) Cardiology: Charles J. Davidson, MD; Diabetology: Mark Molitch, MD (Investigators) Nirat Beohar, MD (Coordinators) Elaine Massaro, MS, RN, CDE, Lynne Goodreau, RN, Fabiola Arroyo, CCT Na Homolce Hospital, Prague, Czech Republic, (Clinical Site): (Principal Investigators) Cardiology: Petr Neužil, MD, PhD, Lenka Pavlíĉková, MD; Diabetology: Štĕpánka Stehlíková, MD (Investigators) Jaroslav Benedik, MD (Coordinator) Liz Coling University of Ottawa Heart Institute/Ottawa Hospital-Riverside Campus, Ottawa, Canada, (Clinical Site): (Principal Investigators) Cardiology: Richard Davies, MD, Christopher Glover, MD, Michel LeMay, MD, Thierry Mesana, MD; Diabetology: Teik Chye Ooi, MD, Mark Silverman, MD, Alexander Sorisky, MD (Coordinators) Colette Favreau, RN, Susan McClinton, BScN New York Medical College/Westchester Medical Center, Valhalla, NY (Clinical Site): (Principal Investigators) Cardiology: Melvin Weiss, MD; Diabetology: Irene Weiss, MD (Investigators) Leo Saulle, MD, Harichandra Kannam, MD (Coordinators) Joanne C. Kurylas, RN, CDE, Lorraine Vasi, RN University, Atlanta, GA (Clinical Site): (Principal Investigators) Cardiology: John Douglas Jr., MD, Ziyad Ghazzal, MD, Laurence Sperling, MD, Spencer King, III, MD, Diabetology: Priya Dayamani, MD, Suzanne Gebhart, MD (Investigators) Sabreena Basu, MD, Tarek Helmy, MD, Vin Tangpricha, MD, PhD (Coordinators) Pamela Hyde, RN, Margaret Jenkins, RN, CDE, CCRC, Barbara P. Grant, CVT Washington Hospital Center/Georgetown University Medical Center, Washington, DC (Clinical Site): (Principal Investigators) Cardiology: Kenneth Kent, MD, William Suddath, MD; Diabetology: Michelle Magee, MD (Coordinators) Patricia Julien-Williams, CNP, Vida Reed, RN, CDE Carine Nassar, RD, MS, CDE Québec Heart Institute/Laval Hôpital, Sainte-Foy, Canada (Clinical Site): (Principal Investigators) Cardiology: Gilles Dagenais, MD; Diabetology: Claude Garceau, MD (Coordinator) Dominique Auger, RN University of British Columbia/Vancouver Hospital, British Columbia, Canada (Clinical Site): (Principal Investigators) Cardiology: Christopher Buller, MD; Diabetology: Tom Elliott, MBBS, (Investigators) Krishnan Ramanathan MBChB, Donald Ricci, MD (Coordinators) Rebecca Fox, PA, MSc, Daniela Kolesniak, MD NYU School of Medicine, New York, NY (Clinical Site): (Principal Investigators) Cardiology: Michael Attubato, MD, Frederick Feit, MD; Diabetology: Stephen Richardson, MD (Investigators) Ivan Pena Sing, MD, James Slater, MD (Coordinators) Angela Amendola, MS, PA-C, RD, CDE, Bernardo Vargas, BS Lahey Clinic Medical Center, Burlington, MA, (Clinical Site, Vanguard Site): (Principal Investigators) Cardiology: Nicholas Tsapatsaris, MD, Bartholomew Woods, MD; Diabetology: Gary Cushing, MD (Investigators) Martin K. Rutter, MD, Premranjan Singh, MD (Coordinators) Gail DesRochers, RN, Gail Woodhead, RN, Deborah Gannon, MS, Nancy Shinopulos Campbell, RN University of Virginia, Charlottesville, VA (Clinical Site): (Principal Investigators) Cardiology: Michael Ragosta, MD, Ian Sarembock, MD, Eric Powers, MD; Diabetology: Eugene Barrett, MD (Coordinators) Linda Jahn, RN, MEd, Karen Murie, RN University of Minnesota/Minnesota Veterans Research Institute, Minneapolis, MN (Clinical Site): (Principal Investigators) Cardiology: Gladwin Das, MB, BS, MD, Gardar Sigurdsson, MD, Carl White, MD; Diabetology: John Bantle, MD (Investigator) J. Bruce Redmon, MD (Coordinator) Christine Kwong, MPH, RD, CDE St. Luke's/Roosevelt Hospital Center, New York, NY (Clinical Site): (Principal Investigators) Cardiology: Jacqueline Tamis-Holland, MD; Diabetology: Jeanine Albu, MD (Investigators) Judith S. Hochman, MD, James Slater, MD, James Wilentz, MD (Coordinators) Sylvaine Frances, PA, Deborah Tormey, RN University of Florida, Gainesville, FL (Clinical Site): (Principal Investigators) Cardiology: Carl Pepine, MD, Karen Smith, MD; Endocrinology: Laurence Kennedy, MDFRCP (Coordinators) Karen Brezner, CCRC, Tempa Curry, RN Saint Louis University, St. Louis, MO (Clinical Site): (Principal Investigators) Cardiology: Frank Bleyer, MD; Diabetology: Stewart Albert, MD (Investigator) Arshag Mooradian, MD, (Coordinator) Sharon Plummer, NP University of Texas at Houston, Houston, TX (Clinical Site): (Principal Investigators) Cardiology: Francisco Fuentes, MD, Roberto Robles, MD; Diabetology: Victor Lavis, MD (Investigators) Jaime Gomez, MD, Cesar Iliescu, MD (Coordinators) Carol Underwood, BSN, RN, CCRC, Maria Selin Fulton, RN, CDE, Julie Gomez Ramirez, BSN, RN, Jennifer Merta, MA, Glenna Scott, RN Kaiser-Permanente Medical Center, San Jose, CA (Clinical Site): (Principal Investigators) Cardiology: Ashok Krishnaswami, MD; Diabetology: Lynn Dowdell, MD (Coordinator) Sarah Berkheimer, RN Henry Ford Heart & Vascular Institute, Detroit, MI (Clinical Site): (Principal Investigators) Cardiology: Adam Greenbaum, MD; Diabetology: Fred Whitehouse, MD (Coordinators) Raquel Pangilinan, BSN, RN, Kelly Mann, RN, BSN, CDE Boston Medical Center, Boston, MA (Clinical Site): (Principal Investigators) Cardiology: Alice K. Jacobs, MD; Diabetology: Elliot Sternthal, MD (Investigators) Susana Ebner, MD, Zoran Nedeljkovic, MD (Coordinator) Paula Beardsley, LPN Fletcher Allen Health Care (Vanguard Site), Burlington, VT (Clinical Site): (Principal Investigators) Cardiology: David Schneider, MD; Diabetology: Richard Pratley, MD, William Cefalu, MD, Joel Schnure, MD (Coordinators) Michaelanne Rowen, RN, CCRC, Linda Tilton, MS, RD, DE Jim Moran Heart & Vascular Institute, Fort Lauderdale, FL (Clinical Site): (Principal Investigators) Cardiology: Alan Niederman, MD; Diabetology: Cristina Mata, MD (Coordinator) Terri Kellerman, RN Baylor College of Medicine, Houston, TX Clinical Site: (Principal Investigators) Cardiology: John Farmer, MD; Diabetology: Alan J. Garber, MD, PhD (Investigators) Neal Kleiman, MD (Coordinators) Nancy Howard, RN, BSN, Debra Nichols, RN, Madonna Pool, RN, MSN Duke University, Durham, NC (Clinical Site): (Principal Investigators) Cardiology: Christopher Granger, MD; Diabetology: Mark Feinglos, MD (Investigators) George Adams, MD, Jennifer Green, MD (Coordinators) Bernadette Druken, RN, CCRP, Dani Underwood, MSN, ANP University of Maryland Hospital, Baltimore, MD (Clinical Site): (Principal Investigators) Cardiology: J. Lawrence Stafford, MD; Diabetology: Thomas Donner, MD (Investigator) Warren Laskey, MD (Coordinator) Dana Beach, RN University of Chicago Medical Center, Chicago, IL (Clinical Site): (Principal Investigators) Cardiology: John Lopez, MD; Diabetology: Andrew Davis, MD (Investigators) David Faxon, MD, Sirimon Reutrakul, MD (Coordinator) Emily Bayer, RN, BSN University of Pittsburgh Medical Center, Pittsburgh, PA (Clinical Site, Vanguard Site): (Principal Investigators) Cardiology: Oscar Marroquin, MD, Howard Cohen, MD; Diabetology: Mary Korytkowski, MD (Coordinators) Glory Koerbel, MSN, CDE, Lisa Baxendell, RN, Debbie Rosenfelder, BSN, CCRC, Louise DeRiso, MSN, Carole Farrell, BSN, Tina Vita, RN Washington University/Barnes Jewish Hospital, St. Louis, MO (Clinical Site): (Principal Investigators) Diabetology: Janet McGill, MD; Cardiology: Ronald Krone, MD, Richard Bach, MD (Coordinators) Carol Recklein, RN, MHS, CDE, Kristin M. Luepke, RN, MSN, Mary Jane Clifton Mount Sinai Medical Center, New York, NY (Clinical Site): (Principal Investigators) Cardiology: Michael E. Farkouh, MD, MSc, Michael C. Kim, MD, FACC; Diabetology: Donald A. Smith, MD, MPH (Coordinators) Ida Guzman, RN, ANP, Arlene Travis, RN, MSN, Mid America Heart Institute, Kansas City, MO (Clinical Site): (Principal Investigators) Cardiology: James O'Keefe, MD; Diabetology: Alan Forker, MD, William Isley, MD (deceased) (Investigator) Richard Moe, MD, PhD (Coordinators) Paul Kennedy, RN, Margaret Rosson, LPN, Aimee Long, RN University of Michigan, Ann Arbor, MI (Clinical Site): (Principal Investigators) Cardiology: Eric Bates, MD; Diabetology: William Herman, MD, MPH, Rodica Pop-Busui, MD, PhD (Investigators) Claire Duvernoy, MD, Martin Stevens, MBBCh (Coordinators) Ann Luciano, RN, Cheryl Majors, BSN Johns Hopkins Bayview Medical Center, Baltimore, MD (Clinical Site): (Principal Investigators) Cardiology: Sheldon H. Gottlieb, MD; Diabetology: Annabelle Rodriguez, MD (Coordinator) Melanie Herr, RN Brown University/Rhode Island Hospital, Providence, RI (Clinical Site): (Principal Investigators) Cardiology: David Williams, MD; Diabetology: Robert J. Smith, MD (Investigators), J. Dawn Abbott, MD, Marc J. Laufgraben, MD (Coordinators) Mary Grogan, RN, Janice Muratori, RNP Houston VA Medical Center, Houston, TX (Clinical Site): (Principal Investigators) Cardiology: Gabriel Habib, MD, MS; Diabetology: Marco Marcelli, MD (Investigators) Issam Mikati, MD (Coordinators) Emilia Cordero, NP, Gina Caldwell, LVN New York Hospital Queens/Lang Research Center, Queens, NY (Clinical Site): (Principal Investigators) Cardiology: David Schechter, MD; Diabetology: Daniel Lorber, MD; Nephrology: Phyllis August, MD, MPH (Coordinators) Maisie Brown, RN, MSN, Patricia Depree, PhD, ANP, CDE Wilhelminen Hospital, Vienna, Austria (Clinical Site): (Principal Investigators) Cardiology: Kurt Huber, MD; Diabetology: Ursula Hanusch-Enserer, MD (Investigators) Nelly Jordanova, MD (Coordinators) Dilek Cilesiz, MD, Birgit Vogel, MD St. Joseph Mercy Hospital/Michigan Heart and Vascular Institute and the Ann Arbor Endocrinology and Diabetes, P.C., Ann Arbor, MI (Clinical Site): (Principal Investigators) Cardiology: Ben McCallister Jr., MD; Diabetology: Michael Kleerekoper, MD, Kelly Mandagere, MD, Robert Urbanic, MD (Investigators) James Bengston, MD, MPH, Bobby K. Kong, MD, Andrew Pruitt, MD, Jeffrey Sanfield, MD (Coordinators) Carol Carulli, RN, Ruth Churley-Strom, MSN The Ohio State University Medical Center, Columbus, OH (Clinical Site): (Principal Investigators) Cardiology: Raymond Magorien, MD; Diabetology: Kwame Osei, MD (Coordinators) Cecilia Casey Boyer, RN, MS, CDE Mayo Clinic-Scottsdale, Scottsdale, AZ (Clinical Site): (Principal Investigators) Cardiology: Richard Lee, MD; Diabetology: Pasquale Palumbo, MD (Coordinator) Joyce Wisbey, RN Angiographic Core Laboratory, Stanford University, Stanford, CA: (Principal Investigator) Edwin Alderman, MD (deceased) (Staff) Fumiaki Ikeno, MD, Anne Schwarzkopf (deceased) Biochemistry Core Laboratory, University of Minnesota, Minneapolis, MN: (Principal Investigator) Michael Steffes, MD, PhD (Staff) Maren Nowicki, CLS, Jean Bucksa, CLS ECG Core Laboratory, Saint Louis University, St. Louis, MO (U01 HL061746): (Principal Investigator) Bernard Chaitman, MD (Staff) Jane Eckstein, RN, Karen Stocke, BS, MBA Economics Core Laboratory, Stanford University, Stanford, CA (U01 HL061748): (Principal Investigator) Mark A. Hlatky, MD (Staff) Derek B. Boothroyd, PhD, Kathryn A. Melsop, MS Fibrinolysis Core Laboratory, University of Vermont, Burlington, VT (U01 HL063804): (Principal Investigator) Burton E. Sobel, MD (deceased) (Staff) Michaelanne Rowen, RN, CCRC, Dagnija Neimane, BS Nuclear Cardiology Core Laboratory, University of Alabama at Birmingham, Birmingham, AL (Astellas Pharma US, Inc.): (Principal Investigator) Ami E. Iskandrian, MD (Staff) Mary Beth Schaaf, RN, BSN Diabetes Management Center, Case Western Reserve University, Cleveland, OH: (Director) Saul Genuth, MD (Staff) Theresa Bongarno, BS, Hypertension Management Center, Lahey Clinic Medical Center, Burlington, MA: (Codirector) Richard Nesto, MD Hypertension Management Center, New York Hospital Queens, Queens, NY: (Codirector) Phyllis August, MD, MPH (Staff) Karen Hultberg, MS Lifestyle Intervention Management Center, Johns Hopkins Bayview Medical Center, Baltimore, MD: (Codirector) Sheldon H. Gottlieb, MD Lifestyle Intervention Management Center, St. Luke's/Roosevelt Hospital Center, New York, NY: (Codirector) Jeanine Albu, MD (Staff) Helene Rosenhouse-Romeo, RD, CDE Lipid Management Center, University of Pittsburgh, Pittsburgh, PA: (Director) Trevor J. Orchard, MBBCh, MMedSci (Staff) Georgia Pambianco, MPH, Manuel Lombardero, MS Safety Officer, North Canton, OH: Michael Mock, MD (deceased) Operations Committee: (Chair) Robert L. Frye, MD (Members) Maria Mori Brooks, PhD, Patrice Desvigne-Nickens, MD, Abby Ershow, ScD, Saul Genuth, MD, Suzanne Goldberg, RN, MSN, David Gordon, MD, PhD, Regina Hardison, MS, Teresa L. Z. Jones, MD, Sheryl Kelsey, PhD, Richard Nesto, MD, Trevor Orchard, MBBCh, MMedSci, Dina Paltoo, PhD, MPH, Yves Rosenberg, MD, MPH Morbidity and Mortality Classification Committee (MMCC): (Chair) Thomas Ryan, MD (Cochair) Harold Lebovitz, MD (Members) Robert Brown, MD, Gottlieb Friesinger, MD, Edward Horton, MD, Jay Mason, MD, Renu Virmani, MD, Lawrence Wechsler, MD Data and Safety Monitoring Board (DSMB): (Chair) C. Noel Bairey-Merz, MD (former Chair) J. Ward Kennedy, MD (deceased) (Executive Secretary) David Gordon, MD, PhD (Members) Elliott Antman, MD, John Colwell, MD, PhD, Sarah Fowler, PhD, Curt Furberg, MD, PhD, Lee Goldman, MD, Bruce Jennings, MA, Scott Rankin, MD.
